# Development of iGET Living, a Digital Graded Exposure Intervention for Youth With Chronic Pain: Multiphase User-Centered Design and Pilot Study

**DOI:** 10.2196/89206

**Published:** 2026-04-17

**Authors:** Lauren E Harrison, Sarah N Webster, Ellison S Choate, Dylan Mayanja, Nicole Jehl, Beth D Darnall, Jennifer N Stinson, Marianne Bonnert, Maria Lalouni, Rikard K Wicksell, Laura E Simons

**Affiliations:** 1 Biobehavioral Pediatric Pain Lab, Department of Anesthesiology, Perioperative and Pain Medicine, Stanford Medicine, Palo Alto, CA, 94131, United States, 1 650-498-2486; 2 Lawrence S. Bloomberg Faculty of Nursing, University of Toronto, Toronto, ON, Canada; 3 Child Health Evaluative Sciences, Research Institute, Hospital for Sick Children, Toronto, ON, Canada; 4 Centre for Psychiatry Research, Department of Clinical Neuroscience, Karolinska Institutet, Stockholm, Sweden; 5 Health Care Services Stockholm County, Center for Epidemiology and Community Medicine, Stockholm, Hungary; 6 Pain Clinic, Saint Göran Hospital, Stockholm, Sweden

**Keywords:** Pediatric pain, exposure treatment, digital intervention, implementation, user-centered design

## Abstract

**Background:**

Pediatric chronic pain affects up to one-third of youth and is associated with significant disruptions in social, emotional, and behavioral functioning. Although behavioral treatments are effective, access remains limited due to geographic, financial, and systemic barriers. Digital behavioral health interventions offer a promising solution, but many lack user-centered design, iterative refinement, and implementation-informed development strategies that support usability and scalability.

**Objective:**

This study aimed to develop and iteratively refine iGET Living, a digital graded exposure intervention for youth with chronic pain, using a combined user-centered and implementation-informed framework, and to evaluate its preliminary acceptability, feasibility, and user-perceived success.

**Methods:**

Guided by the Consolidated Framework for Implementation Research (CFIR) and the mHealth (mobile health) Agile Development and Lifecycle model, intervention development proceeded through 3 phases. Phase 0 translated an evidence-based in-person graded exposure treatment (GET Living) into an initial digital prototype. Phase 1 involved iterative user-centered refinement across 3 cycles of qualitative development sessions with youth with chronic pain (n=15), incorporating think-aloud usability testing, Likert-rated feedback, and rapid qualitative analysis mapped to CFIR constructs to guide real-time modifications to content, design, and functionality. Phase 2 piloted the refined intervention with a new sample of youth (n=38, n=30 completers) recruited from a tertiary pediatric pain clinic to evaluate feasibility, acceptability, treatment credibility and expectancy, and user-perceived functional improvements. Quantitative outcomes were summarized descriptively, and qualitative exit interview data were analyzed using rapid qualitative analysis.

**Results:**

Across development cycles, youth feedback informed substantive refinements to the intervention, including reducing text density, incorporating animated educational videos, enhancing interactive features, and improving navigation and layout. These changes resulted in progressive improvements in clarity, satisfaction, and acceptability across prototypes. In the Phase 2 pilot study, participants reported moderate-to-high treatment credibility (mean of 19.71 out of 30) and expectancy (mean of 17.96 out of 30), as well as high satisfaction (mean of 46.12 out of 60). Acceptability ratings across domains of the Theoretical Framework of Acceptability were favorable. Qualitative exit interviews highlighted the interventions’ perceived role in helping youth re-engage in valued activities.

**Conclusions:**

Using a combined CFIR and agile development approach, iGET Living emerged as a feasible, acceptable, engaging digital graded exposure intervention for youth with chronic pain. These findings highlight the value of integrating implementation frameworks and participatory design early in digital intervention development and support further evaluation in a preliminary efficacy trial.

## Introduction

Pediatric chronic pain is a significant health problem, affecting 20%‐35% of children and adolescents worldwide [1-3], with many experiencing significant disruptions to multiple domains of functioning (eg, social, emotional, and behavioral) [4-6]. Despite robust evidence for behavioral health interventions targeting youth with chronic pain [7], access-to-care barriers (eg, shortage of services outside of urban areas, high treatment-related costs, long provider waitlists) significantly limit accessibility [8,9]. Digital behavioral health interventions offer solutions to these barriers, with existing research supporting their effectiveness, scalability, and capacity to increase access to evidence-based treatment [10-14]. Notably, however, despite great potential and the proliferation of research [13,15-24], only 28% of internet or smartphone app tools are disseminated adequately or in a timely manner, often due to limited sustainability planning, lack of user-centered refinement, and technical constraints [25].

Recent meta-analyses and scoping reviews underscore the growing evidence base and ongoing challenges in developing, evaluating, and implementing sustainable digital interventions for chronic pain [26-28]. Digital interventions developed within academia are often theoretically rigorous but may lack user-centered design features, agile iteration, and technical robustness, limiting uptake and sustainability [10,29]. Another challenge is the limited application of product design and software development principles to digital health research [30]. For example, academic digital interventions often rely on static, text-heavy content, limited interactivity, and minimal iterative usability testing, reflecting constraints in technical expertise, funding, and access to dedicated development teams [10,29].

To bridge the gap between intervention development and sustainable clinical use, recent studies emphasize the need for frameworks that integrate implementation science with user-centered digital development [27,31,32]. The Consolidated Framework for Implementation Research (CFIR) [33] provides a structured approach for identifying multilevel determinants that influence the adoption, implementation, and maintenance of interventions. In parallel, the mHealth (mobile health) Agile Development and Lifecycle model [34] emphasizes iterative, user-centered design, rapid prototyping, and mixed method evaluation. Although independent frameworks, they are complementary: CFIR highlights determinants of implementation (ie, intervention characteristics, outer setting, inner setting, individual characteristics, and implementation processes) that guide intervention development and identification of barriers and facilitators to adoption [27,31,32], while the mHealth Agile model provides practical strategies for digital intervention design and refinement [34]. In this study, CFIR was used to guide the identification and organization of implementation-relevant determinants, including intervention characteristics and end user perceptions that informed design decisions, whereas the mHealth Agile Development and Lifecycle model structured the timing and process of iterative development, testing, and refinement across phases. Together, they offer a comprehensive approach for developing interventions that are evidence-based and implementation-ready.

**Figure 1.**
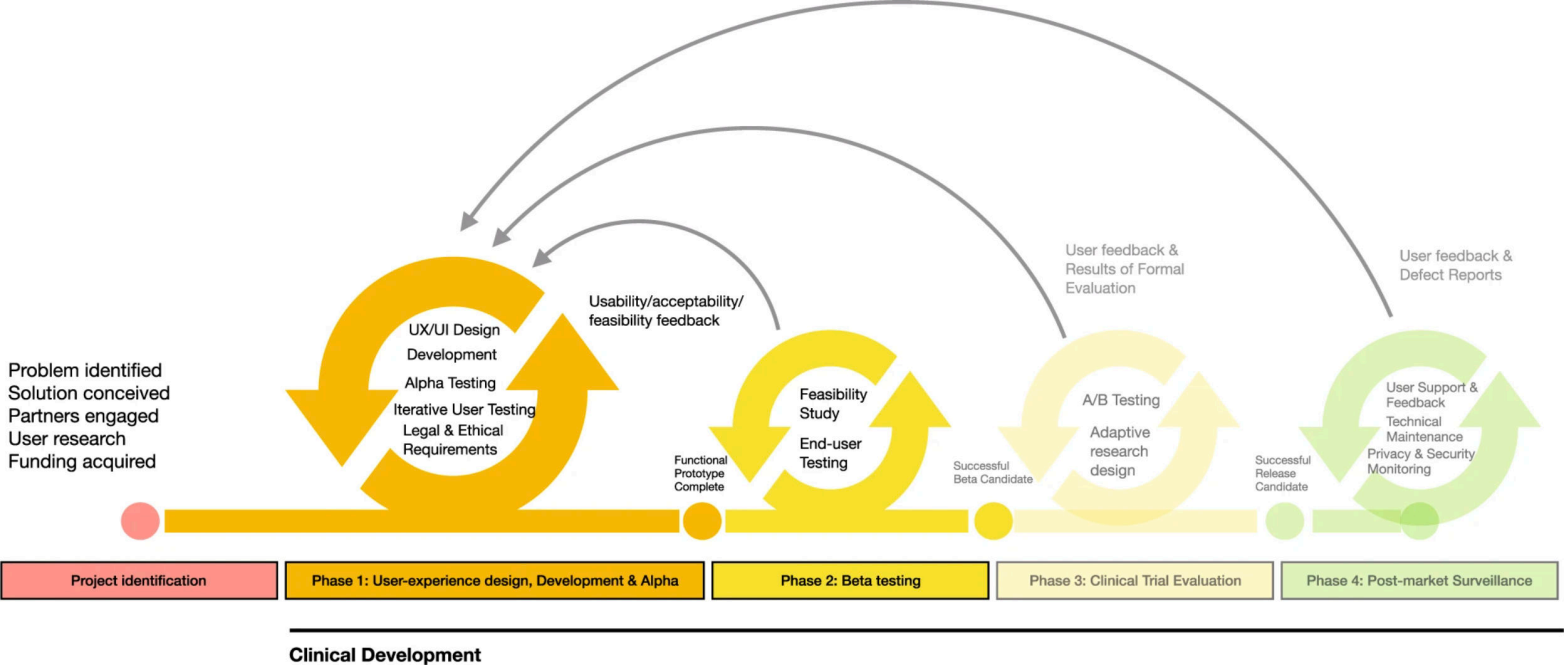
The mHealth (mobile health) agile development and lifecycle model. Reproduced from Harrison et al [35], published under CC BY-NC 4.0.

Despite the growing interest in digital behavioral interventions for chronic pain, relatively few studies have described structured, implementation-informed approaches to intervention development. The objective of this study was to develop and iteratively refine iGET Living, a digital graded exposure intervention for youth with chronic pain, using a combined user-centered and implementation-informed framework, and to evaluate its preliminary feasibility, acceptability, and user-perceived success [35]. The development included 3 phases (Figure 1): project identification and initial prototype development (phase 0), iterative user-centered refinement of the prototype (phase 1), and pilot testing of iGET Living (phase 2). We hypothesized that iterative refinement across phases would result in progressive improvements in clarity, satisfaction, and acceptance of iGET Living, leading to a finalized version of the intervention that was suitable for evaluation in a preliminary efficacy trial.

## Methods

### Study Design

This study is part of a larger, multi-phase project focused on the development, refinement, and pilot testing of iGET Living (NCT05079984). The overall project was guided by 2 complementary frameworks: the mHealth Agile Development and Lifecycle model, which emphasizes rapid, iterative development grounded in user feedback, and the CFIR [33], which provides a structured approach to identify key determinants of successful implementation. The development of iGET Living was reported in alignment with the Guidance for the Reporting of Intervention Development (GUIDED) checklist [36] and the Template for Intervention Description and Replication checklist [37] (Checklist 1).

### Ethical Considerations

All procedures were approved by the Institutional Review Board at Stanford University (Protocol #53323). Informed consent and assent were obtained through a secure, web-based application (REDCap [Research Electronic Data Capture]). All data were collected via REDCap, deidentified, and stored on a secure, cloud-based server. Youth participants in phase 1 received a US $30 Amazon gift code for up to 3 hours of qualitative data collection (user feedback interviews). Youth participants in phase 2 received US $30 gift codes at baseline, discharge, and US $50 gift codes at 3-month follow-up.

### Phase 0: Project Identification and Prototype Development

Phase 0 on translating the core principles of the in-person intervention into a digital prototype. Guided by the principles of agile development, this phase included envisioning a digital solution to overcome access barriers in pediatric pain care, identifying end users, and assembling the multidisciplinary expertise and infrastructure required for digital intervention development. Strategic and operational goals were outlined, and funding was secured to support an iterative design and testing. A microlearning approach was applied to restructure the in-person intervention content into a brief, scalable, digital format. Core components from graded exposure treatment (GET) Living were translated from paper-based worksheets and session activities into digital modules. Content was reorganized for short daily use and supplemented with animated videos and interactive exercises to support engagement in a digital format of the intervention.

### Phase 1: User-Centered Refinement of the iGET Living Prototype

#### Overview

Phase 1 focused on iterative, user-centered refinement of iGET Living. Across 3 cycles of user testing sessions, quantitative and qualitative data on treatment content, clarity, acceptability, layout, and overall engagement were collected. Each cycle informed real-time refinement of modules, navigation structure, and visual presentation.

#### Participants

Participants were recruited from the Pediatric Pain Management Clinic at Stanford Medicine, Children’s Health. To be eligible, youth (1) were between ages 10 and 18 years old, (2) had a diagnosis of chronic (>3 mo) pain, (3) were English literate, and (4) had access to a computer, smartphone, or tablet with internet connection. Review of the literature and previously conducted usability testing studies indicates that prototype refinement is typically achieved within 2‐3 cycles of testing, with ≈5 participants in each cycle [38,39]. Given this, we aimed to enroll 5 youth per interview cycle, with a total of 3 cycles, resulting in a final sample of 15 youth.

#### Procedures

Eligible participants were provided a link to a secure web-based screening form via REDCap, where they left contact information and expressed interest in participation. A research assistant (RA) followed up with a phone call to confirm eligibility, and once confirmed, the RA scheduled a user development session. Consent, assent, and demographic information were obtained via REDCap prior to the user development session. User development sessions were 2 hours long and conducted via Zoom by an RA. During the development session, participants were given access to the digital prototype and were able to view content and pilot features. The development sessions also followed a semistructured interview guide containing Likert-rated and open-ended questions guided by the CFIR Intervention Characteristics domain [33]. Participants were also encouraged to think aloud about likes, dislikes, and difficulties with understanding the content or navigating the prototype. All user development sessions were audio-recorded and stored on a secure cloud-based platform. Data were collected following engagement with each module, as well as at the conclusion of viewing all the content in the prototype. Modules were refined based on quantitative ratings and qualitative data during a series of 3 iterative cycles.

#### Data Analysis

Sample characteristics were examined using descriptive statistics. To facilitate intervention refinement, qualitative data from user development sessions were analyzed using rapid qualitative analysis [40]. Rapid qualitative analysis is a pragmatic and efficient approach to analyzing qualitative data, designed to deliver timely and actionable results. It is often used in intervention development, implementation science, or health services research and prioritizes speed and utility over deep thematic saturation, while maintaining rigor [40-43]. Interview notes were taken from electronic recordings and organized into templated matrices, codes, and subcodes that correspond to the study’s conceptual framework [42,43].

In Phase 1, notes from user development sessions were added to Excel matrices that included codes and subcodes guided by the intervention characteristics domain of the CFIR: relative advantage, adaptability, complexity, and design. This structured approach allowed the research team to efficiently synthesize user perspectives, identify patterns across participants, and implement timely revisions to the intervention content between development cycles. Three researchers independently conducted the qualitative analyses (LEH, SW, and DM).

### Phase 2: Pilot Testing the Prototype

The objective of phase 2 was to pilot the prototype of iGET Living to further evaluate acceptability and feasibility.

#### Participants

An independent sample of participants (ie, did not participate in phase 1) was recruited from the Pediatric Pain Management Clinic at Stanford Medicine, Children’s Health. To be eligible for Phase 2, youth (1) were between ages 10 and 18 years old, (2) had a diagnosis of chronic (>3 mo) pain, and (3) reported at least moderate pain-related disability (Functional Disability Inventory >13) [44]. Eligibility criteria also required English literacy and access to a computer, smartphone, or tablet with an internet connection. Exclusion criteria included significant cognitive impairment or co-occurring medical or psychiatric conditions that could interfere with participation (eg, psychosis active suicidality).

#### Procedures

Eligible participants were provided a link to a secure web-based screening form via REDCap, where they left contact information, completed the Functional Disability Inventory to screen for baseline pain-related disability, and the Patient Health Questionnaire-9, after which a RA followed up with a phone call to confirm eligibility. Eligible participants who selected a 1 or above (ie, several days to nearly every day) on the suicidal ideation question on the Patient Health Questionnaire-9 (“Thoughts that you would be better off dead, or of hurting yourself in some way”) underwent an additional safety assessment screening call with a qualified provider. Once eligibility was confirmed, enrollment procedures were identical to Phase 1. At baseline, youth completed self-report measures assessing demographic characteristics, pain history, pain intensity, pain-related disability, pain-related fear and avoidance, emotional functioning, and quality of life. Following this, the youth were given access to the password-protected platform where the intervention was hosted. All participants were assigned a deidentified username (eg, iGL_1) that they used to sign into the intervention platform. Participants were encouraged to engage with the intervention daily. Given the number of daily modules developed (see Results below), it was estimated that iGET Living would take ≈6 weeks to complete. After the first week of the intervention, youth completed a measure assessing treatment expectancy and credibility. After completing the treatment, the youth completed measures assessing treatment acceptability and satisfaction. Participants also participated in a semistructured exit interview. The self-report assessment battery completed at baseline was repeated at discharge and 3-month follow-up. Assessment of clinical outcomes across timepoints (ie, baseline, discharge, and 3-month follow-up) will be reported separately in a subsequent outcomes-focused manuscript.

#### Measures

##### Demographic Information and Pain History

Youth reported on demographic variables such as age, gender, race, and current grade in school. Youth also reported on the onset and duration of current pain. Average pain severity was assessed using a numerical rating scale [45], ranging from 0 (no pain) to 10 (worst possible pain).

##### Treatment Expectancy and Credibility

To assess expectations for treatment effectiveness, participants completed the Credibility/Expectancy Questionnaire (CEQ) [46] after engaging with iGET Living for one week. The CEQ consists of 6 items rated on a 0 (not at all) to 10 (very much) Likert scale and assesses expectations related to the effects of the intervention across 2 subscales: Credibility (eg, “How confident would you be recommending iGET living to a friend with the same problems?*”*) and expectancy (eg, “By the end of iGET Living, how much do you think your functioning will improve?”). Credibility is calculated by summing items 1‐3, and expectancy by summing items 4‐6. The possible range of scores for each subscale is 0‐30, with higher scores reflecting greater perceived credibility and expectancy.

##### Treatment Satisfaction

Satisfaction with iGET Living was evaluated using the Pain Service Satisfaction Test (PSST) [47]. The PSST is a 20-item measure that assesses participants’ satisfaction with the treatment received and is completed postintervention (eg, “I am satisfied with the treatment I received”). Items are rated on a 3-item scale (0‐3). The PSST yields a total score ranging from 0 to 60, with scores greater than 40 indicating high satisfaction with the treatment. Items on the original PSST were modified slightly to fit with the current intervention.

##### Treatment Acceptability

The Theoretical Framework of Acceptability [48] questionnaire is an 8-item measure that assesses acceptability of an intervention across the following domains: Attitude toward the intervention (“Did you like the intervention?”), Burden (“How much effort did it take to engage with the intervention?”), Ethicality (“How fair were the demands/requirements of the intervention?”), Perceived Effectiveness (“How much do you feel like the intervention has improved your functioning?”), Intervention Coherence (“It is clear to me how the intervention will help me cope with my pain.”), Self-Efficacy (“How confident did you feel about engaging in the intervention?”), Opportunity Costs (“The intervention interfered with my other priorities.”), and General Acceptability (“How acceptable was the intervention to you?”). Items are rated on a 5-point Likert scale ranging from 0 (strongly disagree) to 4 (strongly agree). Scores for each domain are calculated separately. A total score can also be obtained by summing all the items. Higher scores indicated greater acceptability.

##### Semistructured Exit Interview

After completing the intervention, a semistructured interview guide containing Likert-rated and open-ended questions was conducted with each participant by a member of the research team to assess user experiences of engaging with the intervention and overall experience of the treatment (eg, barriers to daily engagement, likelihood of recommending iGET Living to a friend).

### Data Analysis

All quantitative data were analyzed using descriptive statistics in SPSS version 26 [49]. In Phase 2, analyses were guided by the Reflecting and Evaluating construct within the Implementation Process domain of the CFIR [33], which captures end users’ perception of an intervention’s acceptability, feasibility, and perceived effectiveness. Qualitative data were analyzed using rapid qualitative analysis [40,41] and organized into matrices, coded deductively according to the CFIR construct.

## Results

### Phase 0: Prototype Development

The prototype v1.0 of iGET Living was built on the BASS4 platform (Karolinska Institutet eHealth Core Facility). BASS4 is a secure, research-hosted web platform that supports basic interactive features through HTML coding and has been widely used in digital behavioral health intervention research [50-52]. iGET Living v1.0 was organized into 4 primary content domains: Education, Values Clarification and Goal Setting, Activity Exposure, and Planning for Long-term Success. Each domain contained short daily modules (30 modules in total across the 4 domains) that included information and exercises designed for brief (5‐15 min) engagement. This Phase 0 work established a functional prototype of iGET Living, laying the foundation for user-centered refinement in subsequent development cycles.

### Phase 1: User-Centered Refinement of the Prototype

#### Participants

A total of 15 youth were enrolled, with 5 in each iterative cycle, resulting in 3 total cohorts across the 3 iterative cycles. Youth were 15.6 years old on average (range 10‐18 years), with 73.3% assigned female at birth. All youth reported experiencing pain for 3+ years. The average pain rating for the sample was 5.8 (out of 10). Table 1 depicts participant demographic information across cycles. As outlined in Table 2, Phase 1 focused on iterative user-centered refinement through multiple cycles of end user feedback and real-time design iteration.

**Table 1.** Characteristics of the sample for phase 1.

	Cycle 1 (n=5)	Cycle 2 (n=5)	Cycle 3 (n=5)
Age (years)			
Mean (SD)	15.6 (2.2)	16.2 (2.5)	15.0 (3.5)
Range	12‐18	12‐18	10‐18
Gender identity, n (%)			
Cisgender female	5 (100)	4 (80)	3 (60)
Cisgender male	—^a^	1	1 (20)
Nonbinary	—	—	1 (20)
Race/ethnicity, n (%)			
Asian/Pacific Islander	1 (20)	1 (20)	—
Hispanic/Latino	1 (20)	1 (20)	—
White	2 (40)	3 (60)	2 (40)
Multiracial	—	—	2 (40)
Unknown	1 (20)	—	1 (20)
Pain diagnosis, n (%)			
MSK^b^	3 (60)	2 (40)	1 (20)
EDS^c^	—	1 (20)	2 (40)
CRPS^d^	1 (20)	1 (20)	1 (20)
Headache	1 (20)	1 (20)	—
Central sensitization	—	—	1 (20)
Average pain	4.8	6.2	6.5
Average pain duration (year)	3.6	3.8	3.8

a Not applicable.

b MSK: musculoskeletal.

c EDS: Ehlers-Danlos Syndrome.

d CRPS: Complex Regional Pain Syndrome.

**Table 2.** Replicable steps in the user-centered, implementation-informed development of iGET Living.

Phase	Key activities	Data collected	Resulting design decisions
Phase 0	Translate in-person GET^a^ Living into digital prototype	Team consensus; user feedback	Microlearning modules; initial platform
Phase 1 (Cycle 1‐3)	User interviews; think-aloud testing	CFIR-mapped^b^ qualitative responses; Likert ratings	Content simplification; Development of animated videos
Phase 1	Wireframing; platform evaluation	User feedback; Design and development constraints	Migration to Squarespace
Phase 2	Pilot testing	CEQ^c^, PSST^d^, TFA^e^, exit interviews	Final prototype readiness

a GET: graded exposure treatment.

b CFIR: Consolidated Framework for Implementation Research.

c CEQ: Credibility/Expectancy Questionnaire [50].

d PSST: Pain Service Satisfaction Test [51].

e TFA: Theoretical Framework of Acceptability [52].

#### Cross-Cycle Iterative Refinements to Intervention Structure and Content

A summary of end user responses for the Intervention Content domain of the CFIR, along with the resulting action and iteration, is presented in Table 3. Detailed results for each iterative cycle (3 cycles in total) are described below.

**Table 3.** Summary of end user responses and iterations made across cycles mapped onto constructs in the consolidated framework for implementation research (CFIR) intervention domain.

CFIR construct	End user insight	Resulting actions and iterations
Relative advantage	Flexibility in engagement (eg, daily vs every few d) Asynchronous engagement with therapist	Extended treatment length from 6 weeks to 10 weeks Structured weekly emails from therapist
Adaptability		
Platform and Technology Infrastructure	Like the flexibility of using computer or phone	Explicitly acknowledge variability in pacing, encouraged alternative goal setting when necessary, emphasized process over outcome
Tone, Representation, and Inclusivity	Appreciated attempts to include diverse perspectives and use of gender-neutral language Emphasized the importance of acknowledging that not all youth can get back to certain activities in the same way	
Complexity	Initial prototype described as “dense” and like a “textbook” Some modules were described as too text heavy while others were described as clear and concise Requested more examples to explain key concepts Content on connection between pain and mood lacking	Reduced text Streamlined content in modules which resulted in creation of new modules (eg, the module ”Chronic Pain and the Pain Dilemma” became 2 modules: “What is Chronic Pain?” and “The Pain Dilemma” Partnered with Kindea Labs to create animated videos of key concepts Swapped text for figures and images when appropriate
Design		
Visual Layout and Engagement	Youth consistently requested more color, dynamic visuals, and fewer static “text heavy” pages Wanted to be able to visually see their progress through each module and the intervention	Integration of interactive components (eg, being able to type in a text box within a theoretical figure) Added progress bar Integrated color and image to earlier versions when possible
Interactivity and Functionality	Several youth were frustrated when static elements (eg, images) were not clickable Many limitations noted regarding design flexibility, mobile responsiveness, and back-end editing. Many wanted “drag and drop” features and to “see things move” on the web page	Made images clickable where appropriate Content put into “banner” style on webpage (vs list format, for example) Integration of interactive sliders and click-to-enter response boxes Migrated intervention to Squarespace Updated color scheme Inclusion of images

#### Relative Advantage of the Treatment

Overall, youth described the digital delivery of iGET Living as flexible, allowing them to complete sessions at convenient times and frequencies. Youth liked the microsession format, noting it would be easier to “do a quick session here and there” as opposed to going into a doctor’s office, and appreciated that the intervention was mostly self-guided. Youth also reported that daily engagement with iGET Living was feasible. Barriers to daily engagement included physical symptoms (eg, pain, fatigue, dizziness), logistical challenges (eg, other doctors’ appointments, homework), and emotional readiness and motivation to engage.

#### Adaptability of the Treatment

Most participants noted that they would prefer to do iGET Living on a computer, not a smartphone, because the content would be easier to view on a larger screen, and it would be easier to type in module responses on a computer. However, youth expressed the desire for an app-based intervention that would be more accessible on a smartphone (“If it were designed differently, like an app, I would definitely use my phone.”), noting that being able to access the intervention on a smartphone increased flexibility with when and where they would engage in the intervention. Several youth noted no preference (eg, “probably both [computer and smartphone] depending on the day.”

#### Complexity of the Treatment

**Figure 2.**
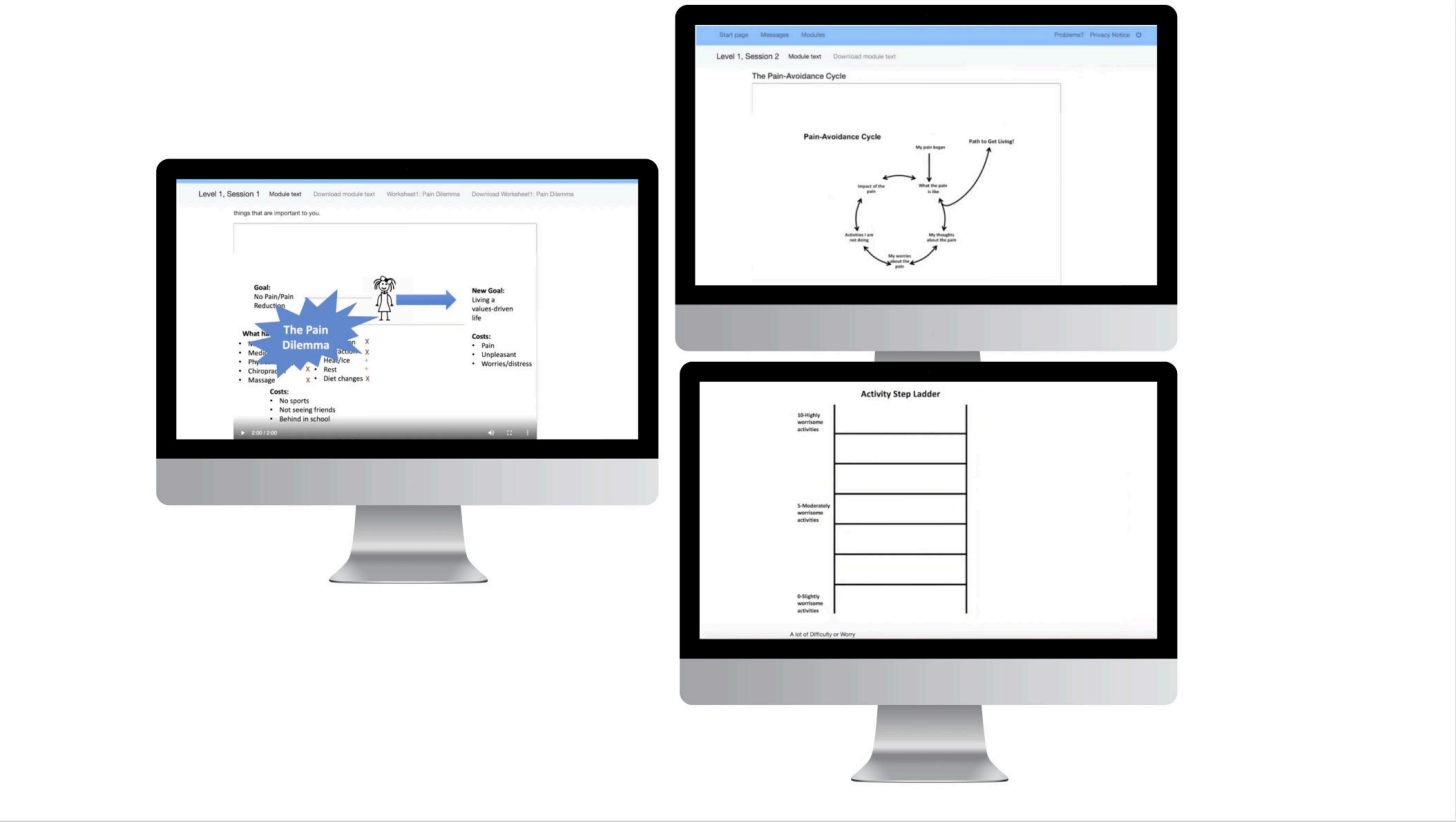
Initial prototype of iGET Living: Voice-over PowerPoint animation and static images of worksheets from the in-person intervention.

Despite rating the modules’ favorability overall (Table 4), youth consistently described v1.0 of iGET Living as “textbook-like,” “too boring,” and “not something I would want to come back to.” Figure 2 shows content from several modules that included a voice-over video made in PowerPoint and examples of images of worksheets used. Youth reported several issues with the use of static images, with one youth sharing, “I want to be able to do something with the content, not just read it.”

**Figure 3.**
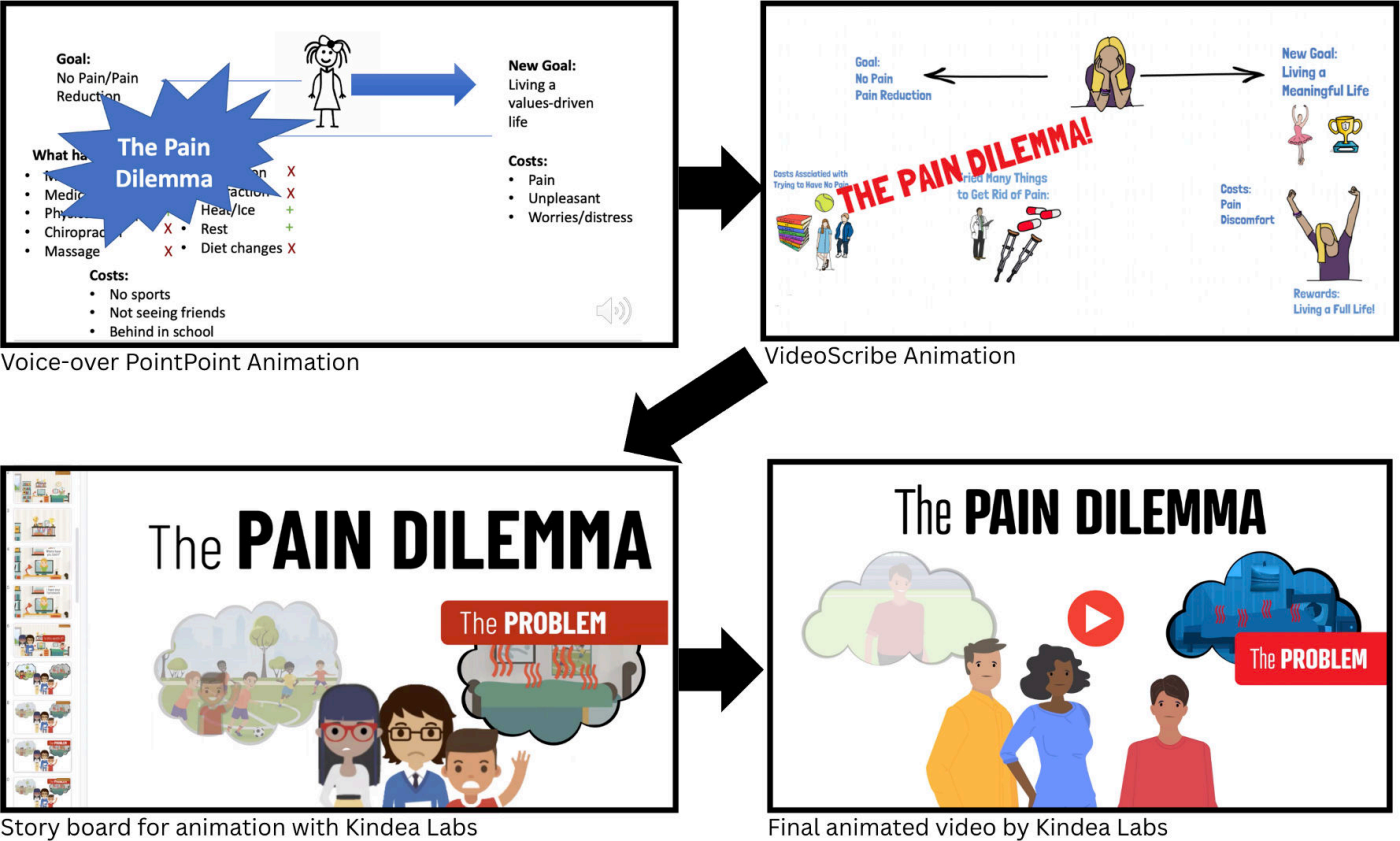
Example of the development process of an animation from voice-over PowerPoint to the final version created in partnership with Kindea Labs.

Significant effort was made to simplify and reduce the text, which resulted in developing animated videos of core treatment concepts. Figure 3 shows the evolution of the Pain Dilemma animation from voice-over PowerPoint to VideoScribe to the final version, which was created in partnership with Kindea Labs. Overall, youth rated the videos very favorably (mean 3.6/4) and reported that the videos were “clear,” “easy to follow,” and “explained things pretty well.” Versions 1.0 and 2.0 of the prototype contained a module titled “Chronic Pain and the Pain Dilemma,” which introduced the distinction between acute and chronic pain and included an exercise based on the concept of creative hopelessness derived from acceptance and commitment therapy [53]. This element was designed to enhance youth readiness to engage with exposure-based activities by reflecting on the limits of avoidance and the potential benefits of values-driven action. In response to end user feedback, this module was divided into 2 shorter modules—What is Chronic Pain and The Pain Dilemma. The What is Chronic Pain module was also expanded to include additional psychoeducation on pain mechanisms (eg, how pain is processed in the brain, metaphors for understanding chronic pain), which had been missing from the initial prototype. The third and final cycle of user-centered interviews reflected that the content was clear and satisfactory (Table 4), as reflected in youth feedback: “The layout is easy to follow,” “Things are explained in an easy-to-understand way, and nothing is too wordy.”

**Table 4.** Likert ratings of treatment content across development cycles. The following prompts were used to assess acceptability of content and design: “Were there features or content that was difficult to learn or understand?” (Difficulty); “Was it clear what you were supposed to do in this module?” (Clarity); and “How satisfied are you with the visual layout?” (Satisfaction). Items were rated on a 5-point Likert scale (0=not at all to 4=very much). Difficulty scores were reverse-scored in this table so that higher scores in each domain indicate a more favorable rating.

Key concepts	Cycle 1 (n=5)			Cycle 2 (n=5)			Cycle 3 (n=5)		
	Difficulty	Clarity	Satisfaction	Difficulty	Clarity	Satisfaction	Difficulty	Clarity	Satisfaction
Education modules									
Chronic pain and the pain dilemma	3.6	3.2	3.2	3.8	4.0	3.6	—^a^	—	—
What is chronic pain?	—	—	—	—	—	—	3.2	4.0	3.8
The pain dilemma	—	—	—	—	—	—	4.0	4.0	3.5
Pain avoidance	2.5	3.6	3.8	4.0	3.8	3.6	3.8	4.0	3.8
Pain, stress, mood	—	—	—	—	—	—	3.5	3.5	3.2
Values and goals	3.7	4.0	3.6	3.0	3.8	3.0	3.7	4.0	4.0
What is exposure?	3.2	4.0	3.8	4.0	2.6	3.4	4.0	3.7	3.7
WILD scale	3.2	3.5	3.2	3.2	2.8	3.2	3.8	3.8	3.8
Experiential exercises									
Building the exposure hierarchy	3.4	4.0	3.6	3.6	3.6	3.4	3.4	3.8	3.6
Building the coping toolkit	—	—	—	—	—	—	4.0	3.8	3.8
Values clarification exercises	3.8	3.4	4.0	3.8	3.8	3.5	3.2	3.6	3.4
Activity exposures	3.4	3.8	3.8	2.2	4.0	4.0	3.6	4.0	3.8
Problem-solving obstacles	—	—	—	—	—	—	4.0	4.0	2.8
Reflecting on accomplishments	3.8	4.0	4.0	3.8	3.2	3.8	4.0	4.0	4.0
Longtime SMART goals	4.0	3.8	3.8	3.8	4.0	4.0	3.8	4.0	4.0
Top lessons learned	4.0	4.0	4.0	3.0	3.6	4.0	3.8	3.8	3.8

a Not applicable.

### Design of the Treatment

**Figure 4.**
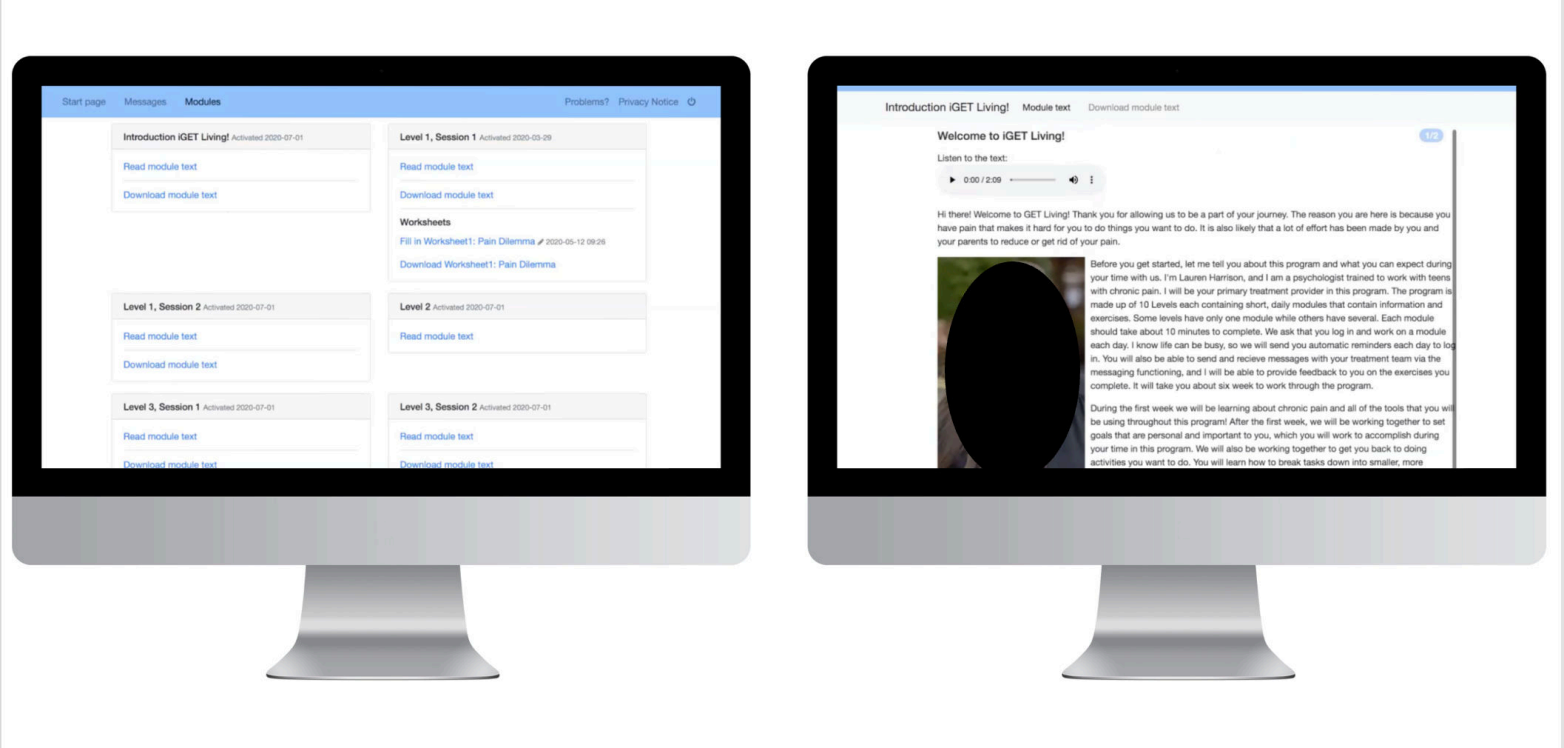
Initial prototype of iGET Living: Homepage and introduction to the intervention.

**Figure 5.**
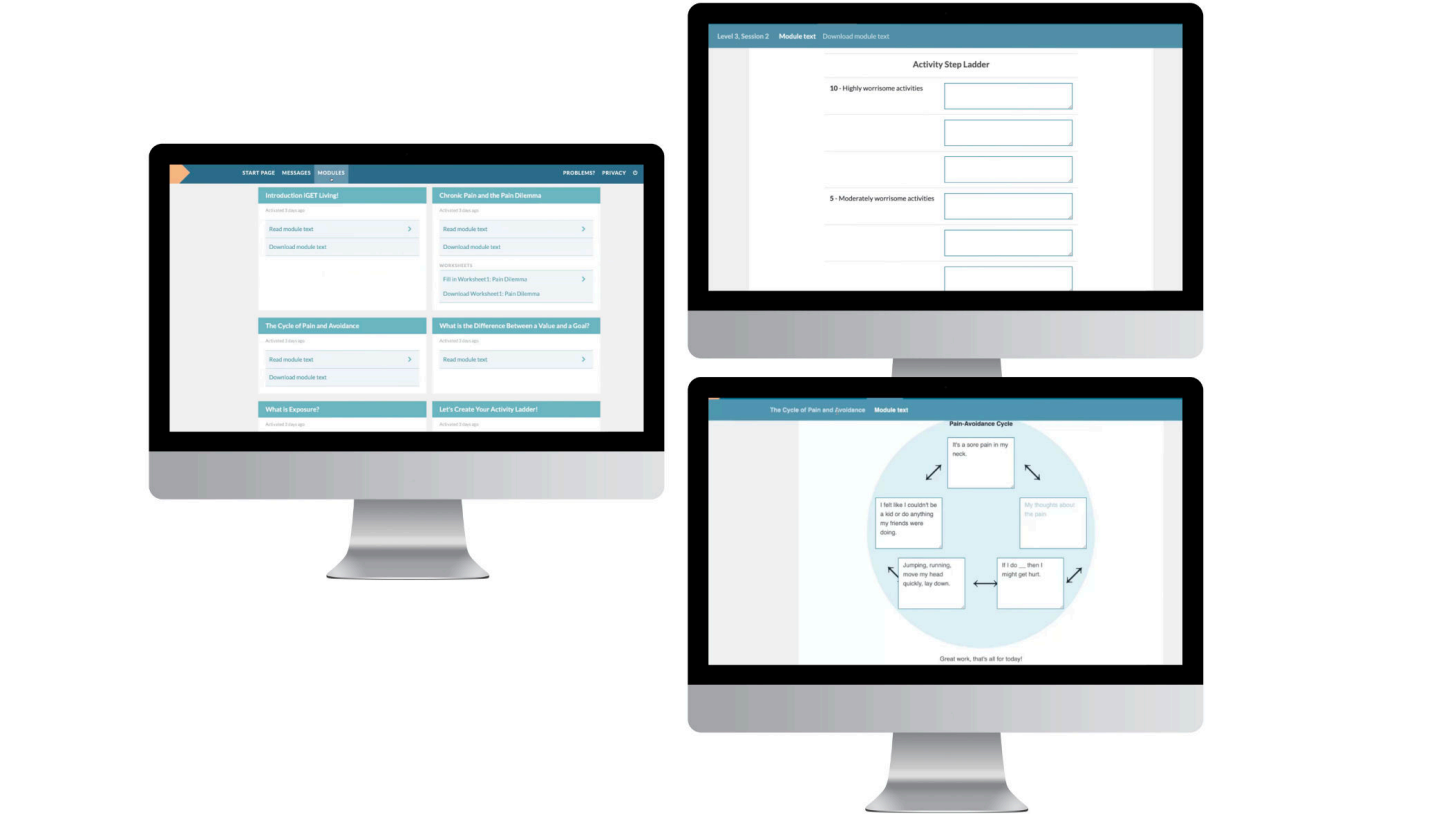
Version 2 of iGET Living reflecting modifications to the home page, integration of the color palette, and the translation of static images into type-in-text features.

Figure 4 shows the main screen and welcome page for the initial prototype (v1.0) developed in Phase 0. Treatment modules were labeled as generic “Levels.” Technical barriers limited the ability to rename these initially (eg, change ‘Level 1’ to ‘Chronic Pain and the Pain Dilemma’), and many youth noted that this was confusing and made the intervention hard to navigate. Updates to the platform allowed for descriptive headers to replace the generic “Level” label with the title of the module. A limited color palette was also integrated. With technological support from the software developers, static images of worksheets were replaced with more interactive, type-in-text box features. Figure 5 shows v2.0 of the prototype, which reflects iterations made in response to user feedback collected during cycle 1.

**Figure 6.**
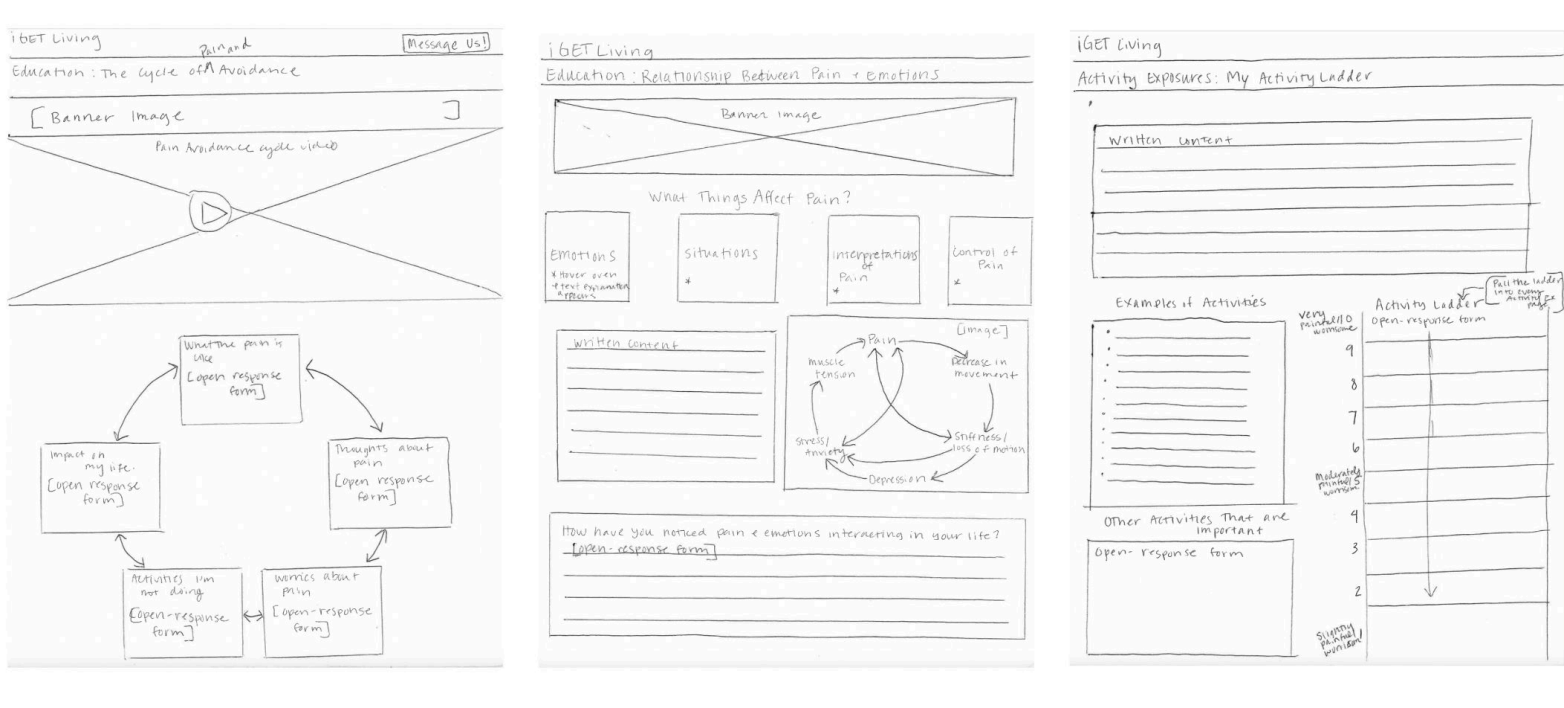
Wireframes of treatment content.

At the conclusion of 2 cycles of interviews (n=10) and several iterations of the intervention content, data suggested that the treatment content of the iGET Living prototype was acceptable and satisfactory to users (Table 3). However, technical and aesthetic limitations of the platform continued to be identified by the users as major barriers to future engagement in the intervention. To further the conceptualization of the intervention that incorporated users’ feedback for technical modifications, wireframes (ie, a screen blueprint that can be used as a visual guide when creating the skeletal framework of a website) were drafted for each component of the intervention (Figure 6). These provided a way to sketch out the ideal layout, organization, and technical features of the intervention and facilitated concrete discussions with software developers regarding desired modifications to the existing digital platform.

**Figure 7.**
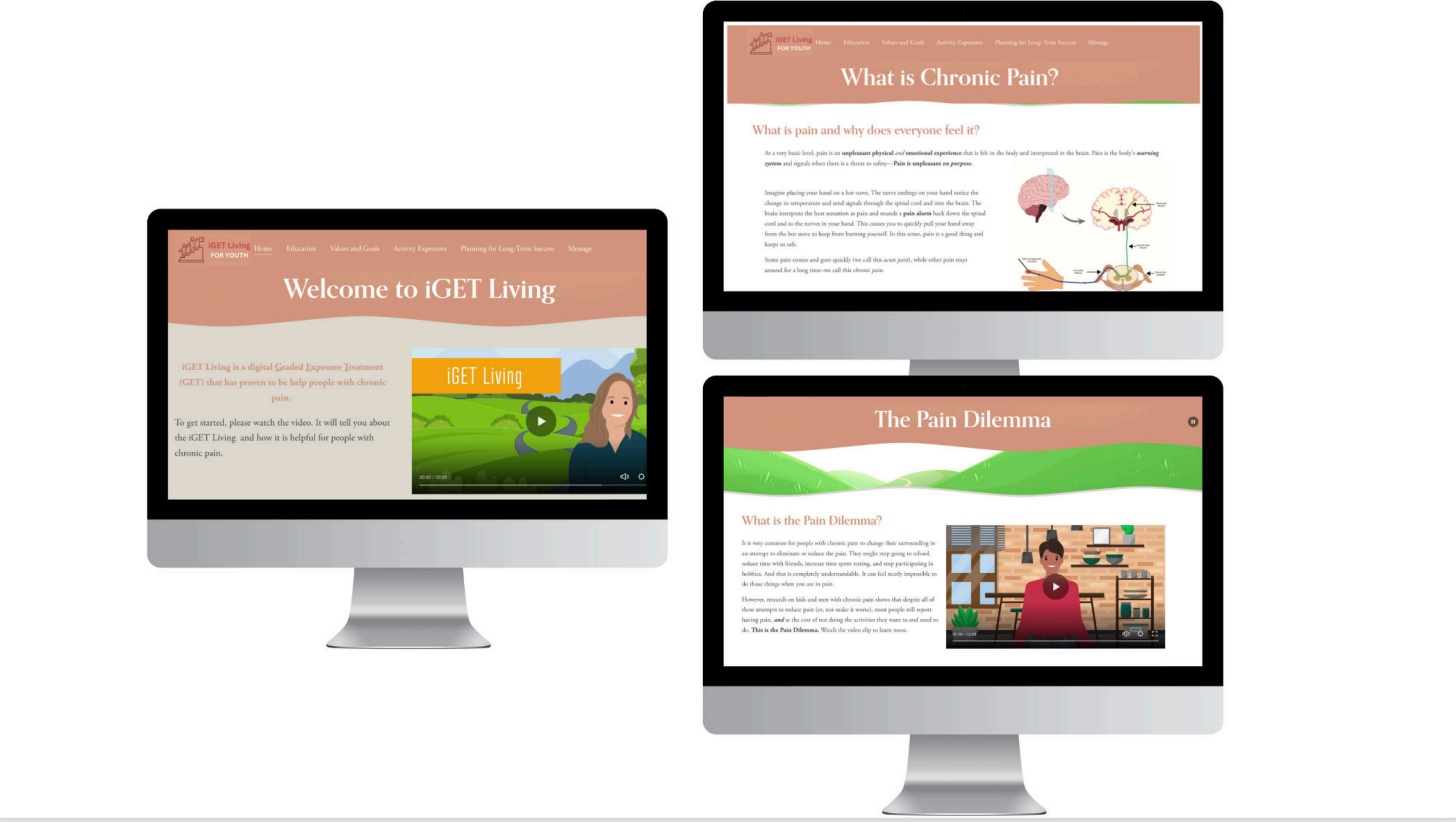
The final prototype of iGET Living is ready for beta testing.

Significant time and costs associated with making changes to the current platform presented barriers, and the decision was made to transfer iGET Living to Squarespace [54], a no-code, low-cost platform that contains premade, user-friendly webpage templates to facilitate the design of a website. Additionally, Squarespace has the capability to store nonprotected health information (eg, deidentified responses to exercises within intervention modules) within the platform. Canva [55], an online graphic design tool for making presentations, was used to create visual content (eg, images, buttons, banners), which was easily uploaded into Squarespace. Using the wireframes as a guide, our research team quickly built a new iGET Living prototype on the webpage Squarespace. Additional edits, including adjusting the spacing of content on the webpage, integration of a progress bar, and inclusion of more images, were made to v3.0 of iGET Living, resulting in a finalized prototype ready for beta testing (Figure 7). iGET Living v3.0 consisted of 4 main modules: Education, Values Clarification and Goal Setting, Activity Exposures, and Planning for Long Term Success. Each module contained self-guided microsessions (30 total across all 4 modules) designed for brief (5‐15 min) daily engagement (Table 5).

**Table 5.** Reflecting and evaluating the construct of the Consolidated Framework for Implementation Research (CFIR) (n=30) following pilot testing (Phase 2) of iGET Living. Items were rated on a 5-point Likert scale (0=not at all to 4=very much).

Question	Mean rating (range)	Participant quotes
How much did you like iGET Living?	3.6 (3-4)	“I liked that there was a lot of time dedicated to the activity exposures—you need a lot of time and practice to work on this.” [iGL_7] “The idea of exposures can be intimidating but I liked that iGET Living focused on baby steps.” [iGL_14]
Do you think iGET Living helped you get back to doing the things you need to/want to do?	3.8 (3-4)	“It gave me some good strategies to try and taught me about pain which was cool. It did not improve my pain, but it got me back to doing things and had a positive impact.” [iGL_34]
How often do you feel like you were able to engage in iGET Living daily?	2.5 (1-4)	“Some days I just really didn’t want to do it, or I would forget or get busy. If I was exhausted after doing something like school, I didn’t want to come home and do this right away.” [iGL_1] “Debilitating pain, bad vertigo, schoolwork, and other doctors’ appointments…this made it hard to do every day.” [iGL_6] “I would sometimes just forget or wouldn’t be in the mood. It takes a lot of initiative.” [iGL_12]
How likely would you be to recommend iGET Living to a friend?	3.3 (1-4)	“I would tell them to do it—it will be beneficial one way or another.” [iGL_27]
How would you describe the purpose of iGET Living?	—^a^	“The purpose is to overcome the increasing pain that prevents kids from getting back to the things they love.” [iGL_3] “To help people with chronic pain get back to their activities.” [iGL_9] “A short 6-week program that can help you develop more strategies and help you make more goals that are more achievable.” [iGL_7]
What would you like to add to iGET Living that would help you better manage your pain?	—	“More physical exercises and stretches” [iGL_14] “More coping strategies for pain like deep breathing and guided imagery.” [iGL_18] “Make it more accessible and easier to access. Having it be more phone friendly would be good.” [iGL_30] “I would like an app-based intervention that is more accessible on my phone.” [iGL_22]

a Not applicable.

### Phase 2: Pilot Testing the Prototype

#### Participants

**Figure 8.**
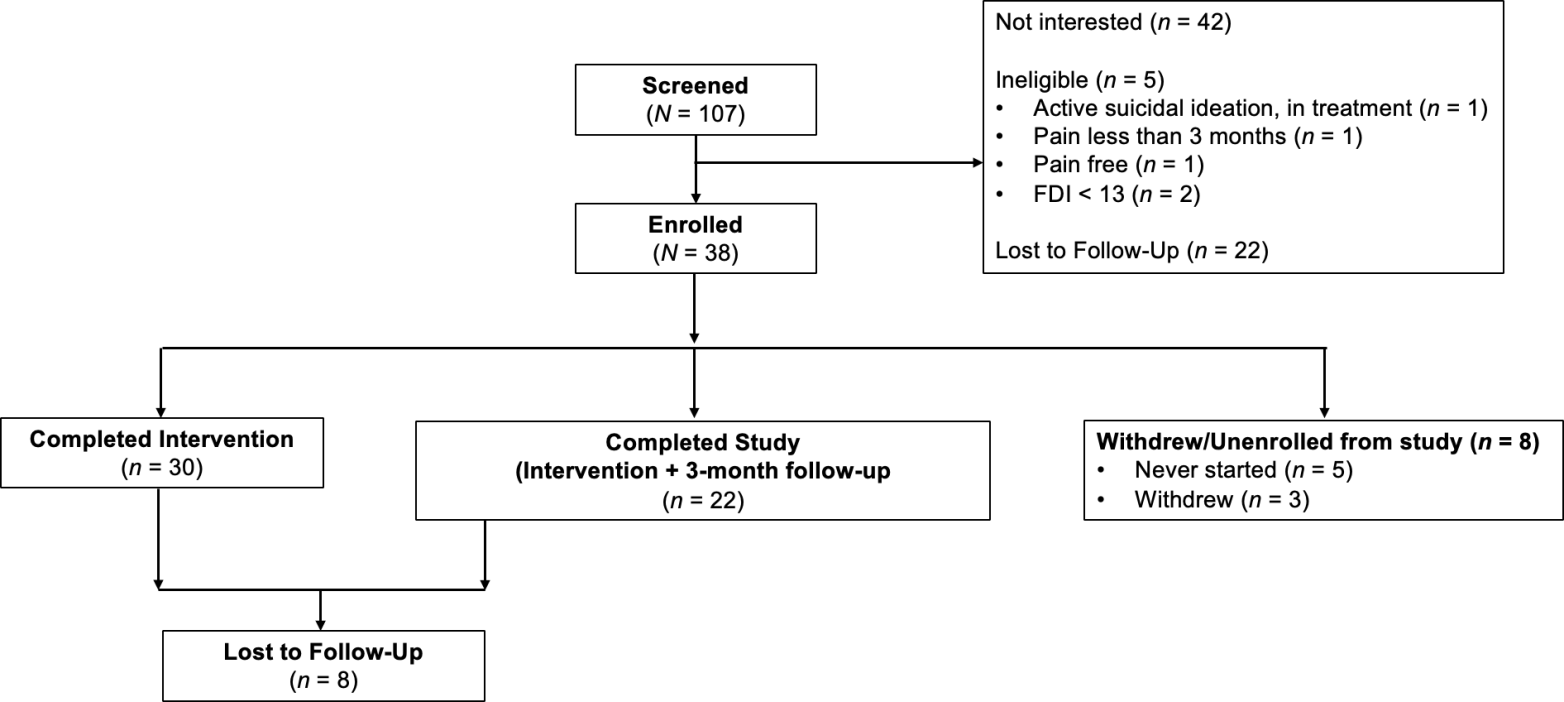
Screening and enrollment flow for Phase 2. FDI: Functional Disability Inventory; LTFU: lost to follow-up.

For Phase 2, 107 youth were screened for eligibility between June 2023 and November 2024 (Figure 8). Of these, 38 youth enrolled in the study (42 were not interested in participating, 5 did not meet eligibility criteria upon further screening, and 5 were lost to follow-up). Of the 38 youth enrolled, 30 youth completed the study procedures (ie, engagement with intervention, completed nondaily and daily assessments). Youth reported an average age of 14.7 (SD 2.4), and 58.3% were assigned female at birth. Youth reported a range of pain diagnoses, including musculoskeletal pain, Ehlers-Danlos Syndrome, Complex Regional Pain Syndrome, and fibromyalgia, and over half (58.2%) of the sample reported experiencing pain for 3+ years. Youth reported an average pain intensity of 6.28 (SD 2.10), with 80.6% of youth reporting experiencing daily pain.

#### End User Perceived Success of the Intervention

On the CEQ, participants reported moderate to high perceptions of iGET Living’s potential effectiveness after engaging with the intervention for one week. Summed scores for the credibility subscale were 19.71 (SD 4.34, range 9‐30) out of a possible 30. Summed scores for the Expectancy subscale were 17.96 (SD 5.83, range 3‐28) out of 30. Treatment satisfaction measured with the modified PSST was also high (mean 46.12*,* SD 11.46).

On the Theoretical Framework of Acceptability questionnaire, participants endorsed favorable ratings across most domains, including attitude toward the intervention (mean 3.32, SD 0.91), ethicality (mean 3.61, SD 0.91), perceived effectiveness (mean 3.35, SD 0.91), intervention coherence (mean 3.32, SD 0.98), and self-efficacy (mean 3.35, SD 0.84). Scores for burden (mean 3.19, SD 0.95) and opportunity costs (mean 2.90, SD 1.22) suggest some participants perceived challenges related to the effort required to engage in the intervention or competing demands. Overall, general acceptability was high (mean 3.81, SD 0.65).

* *Exit interview data, which contained Likert-rated items and open-ended questions, further underscored the acceptability of the intervention (Table 5). Youth described the purpose of iGET Living as helping teens with chronic pain regain function, overcome avoidance, and reconnect with valued activities. They emphasized the importance of the intervention in “getting back to what you used to do,” “helping people with chronic pain get back to the things they enjoy,” and “providing a step-by-step guide to get out of the chronic pain bubble.” Ratings of intervention helpfulness were high, with mean scores of 3.6‐3.8 out of 4. Participants also highlighted areas for improvement, including increasing accessibility (eg, mobile-friendly or app-based formats) and adding more physical exercises. A modified exit interview was conducted with the 3 youth who withdrew from the study. Two of the youth stated that they already knew a lot of the intervention content:

*I’ve done knew a lot of research on my own about my pain so a lot of it, I felt, was just kind of repetitive for me. Like I knew a lot of the stuff already.*

[Y2]

*I feel like it’s all the same information I’ve heard from many, many doctors and specialists*.

[Y17]

The third youth also noted feeling as though it was not the right time for them to engage in this type of intervention:

*I’m looking for a magic pill that’s gonna fix everything I’m dealing with, and I’m not ready to hear all of the other stuff like, that’s just where I’m at. I’m not ready for that. So, it’s not the program—I thought it was great. If I was in a different space, I think I’d definitely do it*.

[Y4]

An outline of the final intervention content can be found in Table 6.

**Table 6.** Final intervention content for iGET Living v3.0 that was piloted in Phase 2.

Module	Daily microsession
Education	What is Chronic Pain? The Pain Dilemma The Cycle of Pain and Avoidance How are Pain, Mood, and Stress Related? What are Values-Based Goals? What is Exposure Treatment
Values and goals^a^	Values clarification and S.M.A.R.T. Goal exercises across the following domains: Friends, Family, Education, Work, Spirituality, Hobbies, Community, Romantic Relationships, Physical Health and Wellbeing, Domain of Your Choice (x3)
Activity exposures	Building the Activity Ladder The W.I.L.D. Scale Building Your Coping Toolkit Activity Exposures (12 in total)
Planning for long-term success	Problem-Solving Obstacles Reflecting on Accomplishments Long-Term Values-Based Goals Top Lessons Learned

a Participants needed to complete five of the 12 values-clarification exercises to be considered “completed.”

## Discussion

### Principal Findings

This study described the iterative, user-centered development and preliminary pilot testing of iGET Living, a digital graded exposure intervention for youth with chronic pain. Guided by the CFIR and the mHealth Agile Development and Lifecycle model, we translated an evidence-based in-person GET into a digital intervention and refined it through multiple cycles of end user feedback. Across development phases, youth participants reported favorable perceptions of credibility, satisfaction, and acceptability, and qualitative feedback highlighted the intervention’s perceived role in supporting functional recovery and re-engagement in valued activities. Together, these findings suggest that integrating implementation science frameworks with user-centered digital development can support the creation of scalable behavioral health interventions for pediatric chronic pain.

The findings are consistent with recent studies showing promise of digital behavioral health interventions for chronic pain [10,11,13,14,32,52,56]. Importantly, our iterative design process aligns with best practices recommended in recent scoping reviews, which emphasize the value of integrating implementation frameworks and user-centered design features early in the development process to support future scalability and uptake [10,27,31].

The study also provides a framework for an agile, iterative development cycle that can be applied to future intervention development. Due to pragmatic constraints commonly encountered in academic digital intervention development, the initial prototype relied on static images, text-heavy content, and basic navigation features to begin translating the evidence-based GET Living protocol into a digital format. This intentionally simplified prototype allowed the research team to prioritize rapid testing of core content and usability before investing in more resource-intensive design features. This approach reflected a pragmatic starting point for development, with the understanding that early iterations would likely be suboptimal in user experience. This anticipated limitation highlights a core challenge in digital health innovation in academic settings—specifically, the difficulty of implementing sophisticated design features without dedicated development teams with substantial funding. Early user feedback played a critical role in identifying necessary improvements and underscored the importance of iterative refinement processes like those outlined in the CFIR and mHealth Agile Development and Lifecycle model. Through user interviews and visual wireframes, we were able to identify key usability challenges, collaborate with developers, and shift platforms to enable more flexible and appealing designs. The final version incorporated high-quality animated videos, interactive features, and concise language to better match youth preferences and developmental needs. As hypothesized, ratings for clarity, satisfaction, and acceptability increased across development cycles and resulted in a finalized version of the intervention suitable for further pilot testing. Feedback from youth end users led to meaningful improvements in clarity, layout, and visual design, as well as enhanced engagement and technical performance, and bolstered findings from prior digital health studies [10,14,56]. These insights underscore the importance of embedding participatory design methods throughout intervention development. The final prototype incorporated structural and aesthetic refinements, expanded content on pain education, and added new modules addressing pain-related mood and stress—all changes directly informed by youth.

This challenge is particularly salient in a digital context where youth are already exposed to health-related information through social media, which is often highly engaging but not always evidence-based. Participatory design does not aim to replicate social media content but rather to identify areas where evidence-based interventions feel misaligned with youths’ experiences, language, or presentation. In this study, youth feedback highlighted when content felt overly dense, repetitive, or insufficiently engaging, prompting iterative refinements to structure, visual presentation, and interactivity while preserving core therapeutic principles.

Notably, our findings emphasize that iterative refinement must extend beyond content to include tone, accessibility, and representation. Youth participants raised concerns about overly clinical language, school-like visuals, and lack of diversity in character design—insights that may have been missed without a structured, participatory design process. This underscores the importance of inclusive developmental frameworks that prioritize relevance and relatability, particularly when designing interventions.

### Limitations and Future Directions

While our sample size was appropriate for alpha testing, it was relatively small and recruited from a single academic medical center. Future work should examine the usability and effectiveness of iGET Living across more diverse clinical and community-based settings. Additionally, although our use of no-code platforms enabled rapid development at low cost, sustaining and scaling the intervention may require partnership with commercial developers or digital health infrastructure. Hybrid effectiveness-implementation designs [27,31] may be particularly valuable for understanding clinical outcomes and contextual determinants of adoption. In the next phase of this project (Phase 3 of the mHealth Agile Development and Lifecycle model), the preliminary efficacy of iGET Living will be evaluated using single-case experimental design methodology [57]. Single-case experimental design offers an approach for rapid evaluation of digital interventions in small cohorts, enabling close examination of individual-level patterns of change. This approach also leverages the intensive, high-frequency data collection possible within digital platforms, aligning well with the iterative nature of agile development.

### Conclusions

In conclusion, this study demonstrates the feasibility of combining evidence-based graded exposure with digital health delivery to address barriers to accessing pediatric pain care. By embedding iterative, user-centered development and implementation frameworks early in the design process, iGET Living represents a promising model for developing scalable, user-informed digital behavioral health interventions. Future work will evaluate the preliminary efficacy of the intervention and examine implementation strategies to support broader dissemination in pediatric pain care settings.

## Supplementary material

10.2196/89206 Checklist 1 GUIDED checklist.

10.2196/89206 Checklist 2 TIDieR checklist

## Abbreviations

CEQ: Credibility/Effectiveness Questionnaire
CFIR: Consolidated Framework for Implementation Research
GET: graded exposure treatment
GUIDED: Guidance for the Reporting of Intervention Development
mHealth: mobile health
PSST: Pain Service Satisfaction Test
RA: research assistant
REDCap: Research Electronic Data Capture
