# Supplementary material for: Development of iGET Living, a Digital Graded Exposure Intervention for Youth With Chronic Pain: Multiphase User-Centered Design and Pilot Study
Source: JMIR Form Res. 2026 Apr 17;10:e89206. doi: 10.2196/89206 (PMC13089625; doi:10.2196/89206)
Supplement: Checklist 1 [file formative-v10-e89206-s001.docx]

| *GUIDED checklist item description*  (Duncan et al., 2020) | Where in the manuscript this is described | Other notes |
| --- | --- | --- |
| 1. Report the context for which the intervention was developed. | *Introduction* (mention of chronic pain population, accessibility issues, the need for digital behavioral interventions) | Also see Harrison et al., 2019 for study protocol. |
| 2. Report the purpose of the intervention development process. | *Introduction,*  *Methods: Study Design)* |  |
| 3. Report the target population for the intervention development process. | *Methods: Study design, Phase 1 Participants, Phase 2 Participants* |  |
| 4. Report how any published intervention development approach contributed to the development process. | *Introduction*,  *Methods: Evidence Base of the Intervention)* |  |
| 5. Report how evidence from different sources informed the  intervention development process. | *Phase 0 – Preparation,*  *Phase 1 - Designing: User-centered design of the digital intervention,*  *Phase 2 - Testing: Piloting the digital intervention* |  |
| 6. Report how/if published theory informed the intervention  development process. | *Introduction*,  *Methods: Evidence Base of the Intervention)* |  |
| 7. Report any use of components from an existing intervention  in the current intervention development process. | *Methods: Evidence Base of the Intervention, Results: Phase 0 Prototype Development* |  |
| 8. Report any guiding principles, people or factors that were prioritised when making decisions during the intervention development process. | *Results: Phase 0 Prototype Development, Phase 1 User-centered Refinement of the Prototype, Phase 2 Pilot Testing the Prototype* |  |
| 9. Report how stakeholders contributed to the intervention  development process. | *Methods: Study Design, Participants (Phase 1 and 2); Results: Phase 1 User-centered Refinement of the Prototype, Phase 2 Pilot Testing the Prototype* |  |
| 10. Report how the intervention changed in content and format from the start of the intervention development process. | *Phase 1 User-centered Refinement of the Prototype, Phase 2 Pilot Testing the Prototype* |  |
| 11. Report any changes to interventions required or likely to be required for subgroups. | *Discussion: Strengths and Limitations, Conclusions* |  |
| 12. Report important uncertainties at the end of the intervention development process. | *Results: Phase 2 Pilot Testing of the Prototype; Discussion* |  |
| 13. Follow TIDieR guidance when describing the developed intervention. | Detailed description of the intervention is provided in the TIDieR checklist (submitted as a supplementary file) |  |
| 14. Report the intervention development process in an open access format. | This paper is reported in an open access format in accordance with the checklist. |  |
