# Supplementary material for: Development of iGET Living, a Digital Graded Exposure Intervention for Youth With Chronic Pain: Multiphase User-Centered Design and Pilot Study
Source: JMIR Form Res. 2026 Apr 17;10:e89206. doi: 10.2196/89206 (PMC13089625; doi:10.2196/89206)
Supplement: Checklist 2 [file formative-v10-e89206-s002.docx]

| *TIDieR checklist item (with item numbers)*  (Hoffmann et al., 2014) | Description |
| --- | --- |
| 1. Brief name:  Provide the name or a phrase that describes the intervention. | iGET Living: Digital Graded Exposure Treatment for Youth with Chronic Pain |
| 2. Why:  Describe any rationale, theory, or goal of the elements essential to the intervention | iGET Living was developed to address the needs in chronic pain by offering an evidence-based, user-centered, and flexible digital behavioral treatment. |
| 3. What:  *Materials*: Describe any physical or informational materials used in the intervention, including those provided to participants or used in intervention delivery or in training of intervention providers. Provide information on where the materials can be accessed (e.g. online appendix, URL)  4.What: | iGET Living contains 41 modules, designed for brief (5-15 minutes) daily engagement. iGET Living was built and delivered via a secure website. |
| *Procedure:* Describe each of the procedures, activities, and/or processes used in the intervention, including any enabling or support activities. | Participants accessed one module per day over a six-to-10-week period. |
| 5. Who provided:  For each category of intervention provider (e.g. psychologist, nursing assistant), describe their expertise, background and any specific training given. | iGET Living is designed to be self-guided. |
| 6. How:  Describe the modes of delivery (e.g. face-to-face or by some other mechanism, such as internet or telephone) of the intervention and whether it was provided individually or in a group. | iGET Living is self-guided and delivered via a secure web platform accessible by computer and mobile device. |
| 7. Where:  Describe the type(s) of location(s) where the intervention occurred, including any necessary infrastructure or relevant features. | iGET Living delivered digitally; participants accessed it remotely at at their own convenience using personal devices (e.g., computer, tablet). |
| 8. When and how much:  Describe the number of times the intervention was delivered and over what period of time including the number of sessions, their schedule, and their duration, intensity or dose. | iGET Living consisted of four main modules: Education, Values Clarification, Activity Exposures, and Planning for Long-Term Success. Each module contained self-guided microsessions (41 total across all four modules) designed for brief (5-15 minutes) daily engagement. |
| 9. Tailoring:  If the intervention was planned to be personalised, titrated or adapted, then describe what, why, when, and how. | iGET Living adopts a user-centered approach that adapts the intervention to end-users' needs and preferences |
| 10. Modifications:  If the intervention was modified during the course of the study, describe the changes (what, why, when, and how). | Changes were made to the prototype based on feedback from focus groups during piloting. |
| 11. How well:  *Planned*: If intervention adherence or fidelity was assessed, describe how and by whom, and if any strategies were used to maintain or improve fidelity, describe them.  12. How well: | N/A |
| *Actual:* If intervention adherence or fidelity was assessed, describe the extent to which the intervention was delivered as planned. | N/A |

1. Duncan, E., et al., Guidance for reporting intervention development studies in health research (GUIDED): an evidence-based consensus study. BMJ open, 2020. 10(4): p. e033516

2. Hoffmann, T.C., et al., Better reporting of interventions: template for intervention description and replication (TIDieR) checklist and guide. Bmj, 2014. 348.
